# Evaluating the Effectiveness of Rural Digital Social Prescribing in Korea: Protocol for a Cohort Study

**DOI:** 10.2196/46371

**Published:** 2023-05-24

**Authors:** Hocheol Lee, Sang Baek Koh, Heui Sug Jo, Tae Ho Lee, Hae Kweun Nam, Bo Zhao, Subeen Lim, Joo Aeh Lim, Ho Hee Lee, Yu Seong Hwang, Dong Hyun Kim

**Affiliations:** 1 Department of Health Administration Software Digital Healthcare Convergence College Yonsei University Wonju Republic of Korea; 2 Yonsei Global Health Center Yonsei University Wonju Republic of Korea; 3 Department of Preventive Medicine Wonju College of Medicine Yonsei University Wonju Republic of Korea; 4 Department of Health Policy and Management Kangwon National University School of Medicine Chuncheon Republic of Korea; 5 Korea Industry Development Institute Gangneung Republic of Korea; 6 Department of Information Statistics Yonsei University Wonju Republic of Korea

**Keywords:** digital health, digital social prescribing, elderly health, community health, digital health intervention

## Abstract

**Background:**

The UK National Health Service (NHS) has introduced a digital social prescribing (DSP) system to improve the mental health of the aging population. In Korea, an ongoing pilot social prescribing project has been implemented for older individuals in rural areas since 2019.

**Objective:**

This research aims to develop a DSP program and to evaluate the effectiveness of the digital platform in rural areas of Korea.

**Methods:**

This study was designed as a prospective cohort method for the development and effectiveness evaluation of rural DSP in Korea. The study divided participants into four groups. Group 1 will continuously implement the existing social prescribing program, group 2 implemented the existing social prescribing program but was switched to DSP in 2023, group 3 newly started the DSP, and the remaining group is the control. The research area of this study is Gangwon Province in Korea. The study is being conducted in Wonju, Chuncheon, and Gangneung. This study will use indicators to measure depression, anxiety, loneliness, cognitive function, and digital literacy. In the future, the interventions will implement the digital platform and the Music Story Telling program. This study will evaluate the effectiveness of DSP using difference-in-differences regression and cost-benefit analysis.

**Results:**

This study was approved for funding from the National Research Foundation of Korea funded by the Ministry of Education in October 2022. The results of the data analysis are expected to be available in September 2023.

**Conclusions:**

The platform will be spread to rural areas in Korea and will serve as the foundation for effectively managing the feelings of solitude and depression among older individuals. This study will provide vital evidence for disseminating DSP in Asian countries such as Japan, China, Singapore, and Taiwan as well as for studying DSP in Korea.

**International Registered Report Identifier (IRRID):**

PRR1-10.2196/46371

## Introduction

Globally, those 65 years or older accounted for 10% of the population in 2022. This number is expected to reach 16% worldwide by 2050 and exceed 20% in all high-income countries [1]. The aging of the global population presents the need to introduce various medical systems.

In the era of aging populations, both the physical and mental health of older individuals should be considered. Depression, solitude, and dementia are emerging social problems [2]. Previous studies have shown that an average of 13%-15% of older individuals 65 years or older experience social solitude, and those 80 years or older and in rural areas experience more social solitude [3,4]. In addition, 6%-13% of older individuals 65 years or older experience loneliness [5,6]. These feelings of social solitude and depression among individuals older than 65 years indicate the vulnerability of mental health in this population, which is highly likely to lead directly to suicide risk and deterioration of physical health.

Due to the current era of aging, the health problems of older individuals cause limitations for the medical personnel and resources of primary health care facilities in local communities. They also lead to financial difficulty for national insurance, which increases the medical burden at the national level [7].

The UK National Health Service (NHS) has introduced a social prescribing system to reduce the workload of primary health caregivers. Currently, the United Kingdom’s primary health caregivers, accounting for one-third of general practitioners, use social prescribing [8]. This system provides a nonclinical social prescribing program through link workers who have been trained in using community resources rather than the clinical medical system. Representative programs of social prescribing include gardening, dance, music therapy, and social activities. Social prescribing is aimed at improving the health of the local community in particular; however, it aims to reduce and minimize the solitude of older individuals 65 years or older; strengthen the local services, the network of the local community, and the efficiency of medical resources; alleviate the burden on primary care; and improve health and welfare services [2].

Previous studies have reported the effects of social prescribing systems on older individuals in various countries. Gardening, nature-friendly activities, and art activities have been found to positively affect the mental health of older individuals [9-11]. In addition, studies identified the effects of social prescribing in increasing self-esteem, improving mental well-being, reducing loneliness, and decreasing the use of medical services among older individuals 65 years or older who felt social isolation and had severe depression [12,13].

In Korea, a pilot project is being implemented for older individuals in rural areas since 2019. The first social prescribing program was performed for 10 weeks; the 10th social prescribing program was implemented in 2022. The social prescribing programs provided were music storytelling, allotment farming, kalimba music therapy, self-help group, and digital literacy strengthening education. These social prescribing programs in Korea reduced the average depression and loneliness of the participating older individuals and enhanced social participation [14-17].

Various experts emphasize the lack of evidence and research on implementing a sustainable system for the social prescribing system as a limitation [18]. The United Kingdom has developed and operated a digital social prescribing (DSP) system to solve these problems using digital platforms. A representative DSP in the United Kingdom is Elemental Software, now under the name Access Health, Support and Social Care, which is mainly responsible for monitoring, observing, and supporting patients from the time when social prescribing is requested. The DSP platform provides health risk analysis, an attendance tracker, health impact measurement, aggregate reporting, befriending services, web-based home resources, grocery collection and pharmacy delivery services, local food bank access, and NHS app library services. As of October 2022, more than 10,000 social prescribing sites have been registered on this DSP platform, and 160 social prescribing service providers use the platform.

Korea has problems similar to the United Kingdom, such as a lack of primary health care personnel and resources in rural areas and insufficient finances for the National Health Insurance [19]. However, Korea is a digital powerhouse among Organisation for Economic Co-operation and Development (OECD) countries with the highest internet penetration rate of 99.9% as of 2021 [20]. The Korean government launched a digital platform in November 2021 and announced that rural areas will be provided with all services through the digital platform. Therefore, it is imperative to develop and operate the existing rural social prescribing model as DSP.

Therefore, this research aims to develop a Korean rural social prescribing program as DSP and to study the possibility of providing and expanding the digital platform to rural areas in Korea, like the Elemental platform in the United Kingdom. Specifically, the first goal is to conduct an economic evaluation by comparing DSP with existing social prescribing programs. Furthermore, it aims to evaluate the effectiveness of reducing depression and solitude when DSP is provided, as the existing social prescribing program does. Finally, it aims to study scenario modeling that extends to Gangwon Province in Korea.

## Methods

### Study Design

This study is designed as a prospective cohort method for the development and effectiveness evaluation of rural DSP in Korea. The study period is designed to be 3 years in total. The study framework was divided into stages by year. The study is designed by dividing participants into four groups. Group 1 will continuously implement the existing social prescribing program, group 2 implemented the existing social prescribing program but was switched to DSP in 2023, group 3 newly started the DSP, and the remaining group is serving as the control.

### Research Area

**Figure 1:**
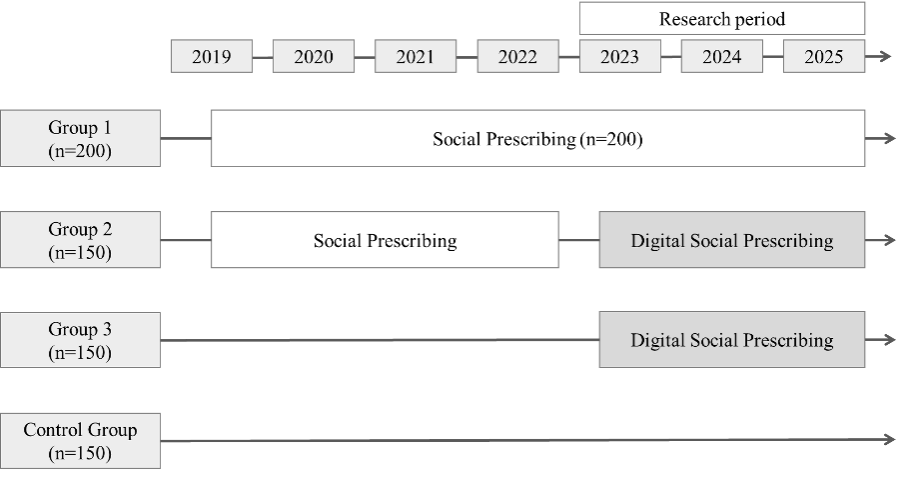
Research framework.

The research area of this study is the Gangwon Province in Korea. The study was conducted in three cities—Wonju, Chuncheon, and Gangneung—which belong to Gangwon Province (Figure 1). Gangwon Province is one of 16 cities/provinces/districts and is among the major regions of Korea. It has a population of 1,555,876, accounting for 2% of the total population of Korea. The older adult population accounts for 22.2% of this population, making Korea a superaged society with the third-highest aging ratio among the 17 cities/provinces/districts in Korea. Chuncheon, Wonju, and Gangneung are three densely populated cities in Gangwon Province with 361,056 people in Wonju, 361,056 in Chuncheon, and 215,322 in Gangneung. In addition, Wonju, Chuncheon, and Gangneung were selected as research areas because the research team works in these areas.

### Study Participants

The participants in this study were older individuals 65 years or older living in Gangwon Province and satisfying the following conditions. Participants had to be older individuals with depression, living alone, and with weak cognitive function. They were recruited after being diagnosed by health care professionals (doctors and nurses) of public health and health service centers. These conditions are the same as those of the existing Korean rural social prescribing model to enable a comparison of the existing model with the DSP model in the future.

### Study Instruments

#### Depression

Older individuals with depression living in rural areas who participate in DSP will be evaluated using the Geriatric Depression Scale Short Form–Korean Version (GDS-K). The GDS is a self-report measurement tool developed by Yesavage [21] to evaluate depression in the older adult population. The GDS-K was developed by modifying and supplementing this measurement tool with expressions that are easy to understand and fit the emotions of older individuals in Korea. Scores range from 0 to 15, with higher scores indicating more severe depressive symptoms. A score of 5 or less is classified as normal, a score of 6-9 as severe depressive symptoms, and a score of 10 or more as depression [22,23].

#### Anxiety

Anxiety will be measured using the Korean version of the Goldberg Short Screening Scale for Anxiety (GSSA) [24,25]. The GSSA consists of nine items on anxiety. The higher the GSSA score, the higher the anxiety level.

#### Loneliness

The Revised UCLA Loneliness Scale will be used to measure social loneliness and solitude [26]. This tool consists of 20 items and is scored out of 20 by responding yes or no. The closer to 20 points, the higher the loneliness. A tool will be used that verifies validity and reliability after translating into Korean [27].

#### Cognitive Function

The Mini-Mental State Examination–Korean Version (MMSE-K) measures overall cognitive function [28]. This tool consists of 30 points, including orientation, memory registration, memory recall, attention and calculation, language skills, and visual composition. A score of 19 or less indicates definitive dementia, a score of 20 to 23 indicates suspected dementia, and a score of 24 or higher is considered normal.

#### Digital Literacy

Digital literacy will measure using the eHealth Literacy Scale (eHEALS) [29]. This tool investigates 23 items in five areas—operational internet skill, information navigation, communicational/social internet skill, creative skill, and mobile internet skill—on a 5-point Likert scale.

### Ethics Approval

The ethics committee of Yonsei University approved this study (REC No. 1041849-202304-SB-073-01). Written informed consent will be obtained from all participants, and the purpose of this study will be carefully explained to them after their permission.

## Results

This study began in October 2022 with support from the National Research Foundation of Korea. The results of the data analysis are expected to be available in September 2023.

### Digital Platform: Electronic Medical Record

Existing social prescribing is performed by patients with doctors, link workers, social prescribing sites, and social prescribing programs. Developing a digital platform allows these to be integrated into one platform. This study will develop a platform through which patients can consult doctors, receive a social prescribing program, communicate with link workers and social prescribing sites, and perform the program. Currently, the Elemental Software program is a representative DSP platform in the United Kingdom [30].

### Music Story Telling Program

The Music Story Telling (MST) program is a social prescribing program based on the theoretical evidence of music therapy and psychological counseling technology. It received a registration patent for service from the Korean Intellectual Property Office in 2013. The MST program is being provided as one of the rural social prescribing programs in Wonju City in Korea, and its effectiveness has been shown through previous research [15].

### Data Analyses

Stata SE 16 (StataCorp) is used for statistical data analysis, and visual analysis will be performed using GraphPad Prism (GraphPad Software, Inc.). We will implement two statistical approaches to understand the effectiveness of rural DSP in Korea. The first tests the effectiveness of DSP, and the second is the economic analysis of DSP.

First, to test the effectiveness of DSP, pre-post comparison for each indicator will be performed using paired *t* test, and the effectiveness of the social prescribing program will be analyzed for each indicator with difference-in-differences regression. In difference-in-differences regression, control variables are input based on the Anderson model to control external factors as much as possible. Statistical significance is analyzed under the significance probabilities of .05, .01, and .001 at the 95% CI. The above statistical method analyzes the intervention effect by dividing it into four research-designed groups.

Second, a cost-benefit analysis is conducted for the economic evaluation of DSP. Cost-benefit analysis is a technique for evaluating input costs and benefits by converting them into monetary values. Costs are estimated as expenses spent on DSP, and benefits are estimated by dividing them into “direct benefits,” which are primary medical costs saved through disease prevention and treatment, and “indirect benefits,” which are incidental results such as improvement in productivity caused by care, prevention, and treatment. The decision indicator for economic evaluation will be finally evaluated based on decision rules using net present value (equation 1) and cost-benefit ratio (equation 2).



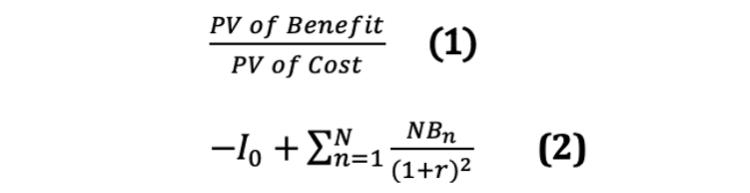



## Discussion

### Expected Findings

As a result of this study, it is expected that an evaluation of the effectiveness of DSP in Korea will be provided. This will include an assessment of the suitability of digital platforms and social prescribing electronic medical records (EMRs) in the Korean context. The study will also aim to propose a model that is appropriate for Korea, taking into account the unique features of the Korean health care system.

Furthermore, by conducting an economic evaluation, this study will provide scientific evidence for expanding the pilot program of social prescribing nationwide in Korea. The results of the economic evaluation will be used to make evidence-based decisions about the allocation of resources and funding for social prescribing initiatives. This will enable policy makers to make informed decisions about how best to invest resources to promote health and well-being in the Korean population.

Overall, this study has the potential to make a significant contribution to the development of social prescribing initiatives in Korea. By providing a comprehensive evaluation of the effectiveness of DSP, as well as proposing a suitable model for digital platforms and social prescribing EMRs, this study will help to ensure that social prescribing initiatives in Korea are evidence based and effective.

### DSP Effect

Among the social prescribing programs implemented in rural Korea, MST, allotment farming programs, and self-help groups have already been identified as effective. If MST is developed as a DSP among these social prescribing programs and provided to older individuals in rural areas, it is expected to be effective in improving participants’ digital literacy in the long-term. A previous study on social prescribing in Korea showed that improved digital literacy in older individuals receiving social prescribing effectively prevents cognitive function deterioration [17].

Globally, social prescribing programs are being converted to DSP programs, and studies on their effects are being published. One study showed that DSP reduced the use of medical facilities, including primary health care [31]. Despite the rapidly aging population structure in rural areas of Korea, a shortage of medical facilities and personnel remains [32]. Hence, DSP can reduce the need for participating older individuals to visit inadequate medical facilities in rural areas of Korea.

### Digital Platform

Korean DSP will be provided by developing and using a digital platform. Although social prescribing programs are continuously being developed and expanded using the digital platform, mainly in the United Kingdom, study cases remain insufficient [33,34]. Elementary Software is a representative example of a digital platform for social prescribing in the United Kingdom [18]. As of August 2022, a cumulative total of 213,757 patients used Elementary Software for social prescribing services [18]. If the study results indicate that DSP is effective, it is expected that digital platforms such as Elementary Software will be widely expanded in Korea. Accordingly, a wide range of big data databases will be automatically collected from the digital platform, and evidence-based DSP studies are expected to be performed.

In terms of the limitations of the existing social prescribing study, there was a significant limitation in data collection to conduct the study on older individuals who participate in social prescribing [35]. Digital platforms will be a cornerstone for developing data-oriented regionally customized programs for older individuals in rural areas participating in social prescribing in the future.

### Social Prescribing EMRs

In Korea, 97.3% of hospitals use EMR systems in line with the development of information and communication technology [36]. The EMR system has advantages in that it makes managing patient data easier. Furthermore, it programmatically protects personal information. DSP also establishes social prescribing EMRs through the digital platform, which is used to record hospitals, link workers, and sites that provide social prescribing in the region for each patient. Data are gathered so that sustainable social prescribing can be performed. These data are expected to be evidence for tracking and managing the patient’s condition.

### Economic Evaluation

Social prescribing programs have been developed, spread, and conducted worldwide, including in Australia, Singapore, Denmark, and Korea, centering on the United Kingdom. A previous study revealed that unnecessary use of primary health care decreased due to social prescribing, thereby reducing unnecessary medical expenses [8]. However, studies on the economic cost-effectiveness of social prescribing programs in the local community are insufficient [31]. There are no cost-effectiveness studies of social prescribing programs in Korea. Therefore, this study plans to conduct an economic evaluation of the social prescribing program in Wonju City, which has been performed since 2019, using cost-effectiveness analysis. The evaluation is expected to prove that the social prescribing program is more cost-effective than the cost of primary health care for older individuals in rural areas in Korea. In addition, an economic evaluation will be carried out cost-effectively when it is developed as a DSP and conducted in rural areas. Accordingly, it will be possible to compare the economic evaluation of existing social prescribing programs and DSP programs, and it is expected to prove the effect hypothesis of digitalized social prescribing.

### Limitations

This study has several limitations. First, Korea has a low level of social awareness about social prescribing programs. Hence, there was a limitation regarding asking study participants and stakeholders for insights on this project. Second, because there is no digital platform for social prescribing, most of the study period was used for technology development, making the study period practically insufficient. Third, the digital literacy of Korean older adults in rural areas was low. Thus, when social prescribing is converted to DSP, it is highly likely that the study participants will have difficulty completing the program.

### Conclusions

As the aging population grows rapidly in Korea, institutional policies using local community resources are needed to reduce the growing burden on primary health care. A review of the current literature shows that social prescribing, spreading worldwide and mainly in the United Kingdom, is effective in improving depression and solitude in older individuals. Therefore, the pilot project for the social prescribing program in rural Korea is being implemented, and its effectiveness has been identified.

However, the existing social prescribing system lacks sustainability due to the limitations of personnel and resources. Therefore, a social prescribing program is being developed using a digital platform. The platform will be spread to rural areas in Korea and will serve as the foundation for effectively managing the feelings of solitude and depression among older individuals. This study will provide vital evidence for disseminating DSP in Asian countries such as Japan, China, Singapore, and Taiwan as well as studying DSP in Korea.

## Abbreviations

DSP: digital social prescribing
eHEALS: eHealth Literacy Scale
EMR: electronic medical record
GDS-K: Geriatric Depression Scale Short Form–Korean Version
GSSA: Goldberg Short Screening Scale for Anxiety
MMSE-K: Mini-Mental State Examination–Korean Version
MST: Music Story Telling
NHS: National Health Service
OECD: Organization for Economic Co-operation and Development

## Data Availability

Data sharing is not applicable to this paper as no data sets were generated or analyzed during this study.
